# Rapamycin: A Bacteria-Derived Immunosuppressant That Has Anti-atherosclerotic Effects and Its Clinical Application

**DOI:** 10.3389/fphar.2018.01520

**Published:** 2019-01-07

**Authors:** Yandong Liu, Futang Yang, Sili Zou, Lefeng Qu

**Affiliations:** Department of Vascular and Endovascular Surgery, Changzheng Hospital Affiliated to the Second Military Medical University, Shanghai, China

**Keywords:** atherosclerosis, cardiovascular disease, inflammation, immune response, autophagy, rapamycin

## Abstract

Atherosclerosis (AS) is the leading cause of stroke and death worldwide. Although many lipid-lowering or antiplatelet medicines have been used to prevent the devastating outcomes caused by AS, the serious side effects of these medicines cannot be ignored. Moreover, these medicines are aimed at preventing end-point events rather than addressing the formation and progression of the lesion. Rapamycin (sirolimus), a fermentation product derived from soil samples, has immunosuppressive and anti-proliferation effects. It is an inhibitor of mammalian targets of rapamycin, thereby stimulating autophagy pathways. Several lines of evidence have demonstrated that rapamycin possess multiple protective effects against AS through various molecular mechanisms. Moreover, it has been used successfully as an anti-proliferation agent to prevent in-stent restenosis or vascular graft stenosis in patients with coronary artery disease. A thorough understanding of the biomedical regulatory mechanism of rapamycin in AS might reveal pathways for retarding AS. This review summarizes the current knowledge of biomedical mechanisms by which rapamycin retards AS through action on various cells (endothelial cells, macrophages, vascular smooth muscle cells, and T-cells) in early and advanced AS and describes clinical and potential clinical applications of the agent.

## Introduction

Atherosclerosis (AS), which is a chronic inflammatory disease, is a major cause of death and a huge economic burden worldwide (Benjamin et al., 2017). AS commonly affects medium-to-large vessels, such as the carotid, coronary and lower extremity arteries. It causes luminal stenosis or obstruction, which can lead to ischemic stroke, acute coronary syndrome, and inferior limb ischemia. The atherogenic mechanism is incompletely understood, but impaired lipid metabolism and an unresolved inflammatory and immune-response state have been implicated (Tall and Yvan-Charvet, 2015). AS is initially characterized by a series of events: Circulating monocytes are recruited into the subendothelial layer in response to various stimuli and differentiate into macrophages. The macrophages ingest excessive amounts of modified LDL to form foam cells (Tabas and Lichtman, 2017). At the advanced stage, the main pathological feature of AS is unresolved immune-inflammation and imbalance between apoptotic-cell formation and phagocytosis (Tabas and Bornfeldt, 2016). Finally, atherosclerotic plaque can be vulnerable, with a necrotic lipid core encompassing recruited inflammatory cells, cellular necrosis and a thin fibrous cap with few VSMCs and secreted extracellular matrix. Thrombosis, a devastating event of AS, then is induced (Niccoli et al., 2014). These activities are carried out by various cell types in the vascular system: VSMCs, macrophages, endothelial cells and T-cells are the main cell types implicated (Deanfield et al., 2007; Lacolley et al., 2017; Tabas and Lichtman, 2017). Therefore, exploration of pharmaceutical methods to target the aberrant action of cellular components in atherosclerotic plaque formation is imperative. However, the main therapeutic medications, i.e., lipid-lowering and anti-platelet agents and thrombolytics, have undesirable side effects. Moreover, these “gold standard” drugs, which have the desirable effect of preventing the end-point events of advanced AS, such as acute thrombosis, do not reverse AS or correct the aberrant cellular activities. In recent years, some new compounds have been explored as potential sources of anti-AS medicines. For example, plant constituents, such as flavonoids, have been demonstrated to confer therapeutic effects on AS (Luo Y. et al., 2017). However, lack of clinical trials designed to examine the clinical effects of such natural products limited their use. On the other hand, there is a growing awareness of the role of Chinese herbal medicine in the treatment of AS, targeting multiple cellular mechanisms (Liu and Huang, 2016). However, compounds included in one herb, even in an extract of one herb, are very complicated; thus, identifying the mechanism of herbs in treating is difficult.

Rapamycin (RAPA), also known as sirolimus, is a fermentation product derived from *Streptomyces hygroscopicus*, which was isolated from a soil sample collected on Easter Island in 1976 (Abraham and Wiederrecht, 1996). The product was initially used in treating carcinoma or organ transplant rejection as an anti-proliferation, immunosuppressive agent (Garcia and Rini, 2007; Chih et al., 2016). Since 2003, SES have been used to treat *de novo* AS and in-stent or graft restenosis through the drug’s anti-AS effects. Also, through *in vitro* and *in vivo* studies, RAPA has demonstrated pleiotropic effects on cell types involved in AS through multiple signaling pathways that mediate anti-inflammatory, anti-proliferation, immunoregulation and lipid-regulatory processes. Above all, RAPA, as an autophagy stimulator, can modulate cellular autophagy, thereby modifying the balance of cellular proliferation and survival (Sanchez-Plumed et al., 2006). An imbalance in these activities is considered a crucial component in the pathogenesis of AS. Therefore, this natural product is a potential candidate for the prevention or treatment of AS.

## Rapamycin

RAPA (C_51_H_79_NO_13_) was initially found as a lipophilic macrolide antibiotic that inhibits the growth of filamentous fungi (Sehgal et al., 1975). Although it does not have powerful antibiotic effects, RAPA has been used as a potent immunosuppressant agent to suppress allograft rejection in heart (Keogh et al., 2004), liver (Trotter et al., 2001), lung (Shitrit et al., 2005), and kidney (Kahan et al., 1998; Cowan and Heizer, 2000; Kahan, 2000) transplantation. RAPA interferes with crucial signal transduction pathways through binding with FK506-binding-protein. It then inhibits the CKI p27^kipl^, thereby retarding cell-cycle progression at the G1/S transition (Abraham and Wiederrecht, 1996).

**FIGURE 1:**
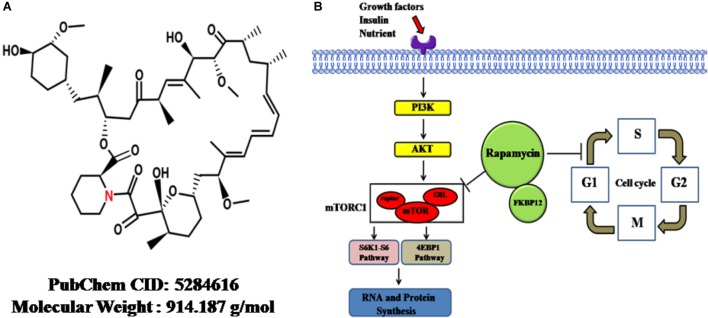
The cellular functions and mechanisms of rapamycin. **(A)** The chemical structure of rapamycin obtained from PubChem database. **(B)** Rapamycin-induced cellular functional changes and mechanisms. Upon reaction with stimulators, such as growth factors, insulin, or adequate nutrients, the PI3K-AKT pathway is driven, followed by the activation of mTORC1. Then, S6K1-S6 and 4EBP-1 pathways, two direct targets of mTORC1, are phosphorylated by activated mTORC1, thereby promoting RNA and protein synthesis. Rapamycin can inhibit the kinase activity of mTORC1, thereby inhibiting synthesis. On the other hand, rapamycin can retard cell-cycle progression at the G1/S transition. mTOR, mammalian target of rapamycin; GβL, G-protein β-subunit like protein; mTORC1, mTOR complex 1; S6K1-S6, ribosomal protein S6 kinase 1-ribosomal protein S6; 4EBP-1, eukaryotic initiation factor 4E–binding protein 1; Atg1, autophagy-related genes; FKBP12, FK-binding-protein-12.

On the other hand, the mTOR, a serine/threonine protein kinase, forms two complexes with distinct proteins: mTORC1 and mTORC2. These proteins are direct targets of RAPA and regulate protein synthesis; cellular proliferation, differentiation and survival; and lipid metabolism (Soltani et al., 2018). The mTORC1 composite consists mainly of mTOR, raptor, and G-protein β-subunit protein (GßL). The ribosomal protein S6K1-ribosomal protein S6 pathway and eukaryotic initiation factor 4EBP-1, as two direct targets of mTORC1, are phosphorylated by mTORC1 after stimulation with growth factors or other factors, thereby stimulating RNA and protein synthesis, cell growth and cell survival (Nemchenko et al., 2011). RAPA inhibits the kinase activity of mTORC1 through binding to the FKBP (FK-binding-protein) 12–rapamycin-binding domain; through this mechanism, cells are differentiated and less proliferative (Tarantino and Capone, 2013). The chemical structure and mechanism of actions were summarized in Figure 1.

## Early Atherosclerosis

Early AS formation is characterized by endothelial-monocyte interaction, foam cell formation and the de-differentiation of VSMCs. In its initial stage of AS, the loss of integrity of the endothelial layer is a requisite for the formation and development of the lesion (Roth et al., 2015). Normal vasoconstriction and relaxation of endothelial cells are requisites for maintaining vascular homeostasis. These activities are largely determined by NO, produced by activation of eNOS (Mineo and Shaul, 2013). Decreased production of NO in endothelial cells, causing excessive vasoconstriction, can contribute to the initiation of AS and in-stent restenosis. Meanwhile, driven by factors such as shear stress, oxidative stress or high glucose concentrations, vascular adhesion molecules, including VCAM-1, ICAM-1, and E-selectin, are induced. Circulating monocytes then are incorporated into the subendothelial layer, where they engulf oxidized lipid and help promote AS (Schmitt et al., 2014; Khodabandehlou et al., 2017). In turn, deposited OX-LDL can increase the expression of adhesion molecules, leading to recruitment of more inflammatory cells.

After being enrolled into the subendothelial layer, monocyte-derived macrophages engorge cholesterol and form foam cells. Accumulation of foam cells results from discrepancy between uncontrolled modified lipid uptake and insufficient efflux (Randolph, 2014). Depending on SRs, including SR-A, SR-BII, CD36, and LOX-1, infiltrated macrophages engulf OX-LDL and form foam cells (Tall and Yvan-Charvet, 2015). On the other hand, the ABCA1 and G1 (ABCG1) modulate cholesterol efflux and retard foam cell formation. This process is mainly regulated by two main nuclear hormone receptors: PPAR-γ and its downstream target, LXRα. OX-LDL can then be absorbed by endothelial cells and macrophages through scavenger or LOX-1 receptors (Kume and Kita, 2004; Akhmedov et al., 2014). Therefore, targeting lipid-laden macrophages and endothelial cells by inhibiting lipid uptake and enhancing cholesterol efflux is an effective method for inhibiting AS formation.

Following the endothelial activation and monocyte recruitment, VSMCs contribute to AS formation. Normally, VSMCs are located in vessel media, with a well-recognized phenotype (“contractile”), functioning as a relaxation and constriction mediator. In pathological conditions, when atherogenic stimuli such as OX-LDL are present, VSMCs take on synthetic, dedifferentiated phenotype. Their migratory and proliferative abilities are activated, i.e., VSMC migration into the intima and hyperplasia and secretion of extracellular matrix, resulting in thickening and stiffness of vessel intima (Farb et al., 2004; Bennett et al., 2016). Suppression of VSMC phenotypic transformation, therefore, may be an effective strategy for retarding AS formation.

## Advanced Atherosclerosis

Advanced AS is characterized by unresolved inflammatory response and excessive cellular apoptosis and death. The inflammatory immune responses, which are dominated by macrophages, are the major contributors to the unresolved inflammation in AS (Tabas and Bornfeldt, 2016). The innate inflammatory response is mainly regulated by TLR4, an immune receptor for detecting damage-associated molecular patterns. On the other hand, the adaptive immune response is mainly mediated by T cells and is modulated principally by mechanisms that, in AS, affect the balance between regulatory and effector T cells. Mechanistically, a skew toward effector T cells other than Treg cells promotes AS, and Treg cells are decreased in AS (Tabas and Lichtman, 2017). Treg cells have anti-inflammatory properties and release TGF-β. In brief, the inflammatory elements in vessels can drive the oxidation of LDL, leading to further formation of foam cells, thus creating a vicious cycle to promote the development of AS. Therefore, decreasing innate and adaptive immune response may be an effective strategy for retarding AS development.

In advanced AS lesions, excessive macrophage or VSMCs-derived foam-cell populations can induce increased inflammatory response and extracellular matrix metalloproteinase and ROS, which cause cellular apoptosis and cell death (Allahverdian et al., 2014). During the process, where excessive stress (oxidative, metabolic, or inflammatory) exists, autophagy becomes impaired, which makes macrophages and VSMCs vulnerable to apoptotic stimuli, resulting in plaque instability (De Meyer et al., 2015). Therefore, the strategy of inhibiting cellular apoptosis and death via enhancing autophagy is logical for preventing plaque formation. Addtionally, senescent VSMCs, which are commonly present in unstable human carotid plaque, have been reported to have impaired self-repairing capacity, which lessens the protective components in plaque (Gardner et al., 2015). Hence, inhibiting cellular senescence is a potent method for maintaining plaque stability.

## Anti-Atherosclerotic Effects of Rapamycin

**FIGURE 2:**
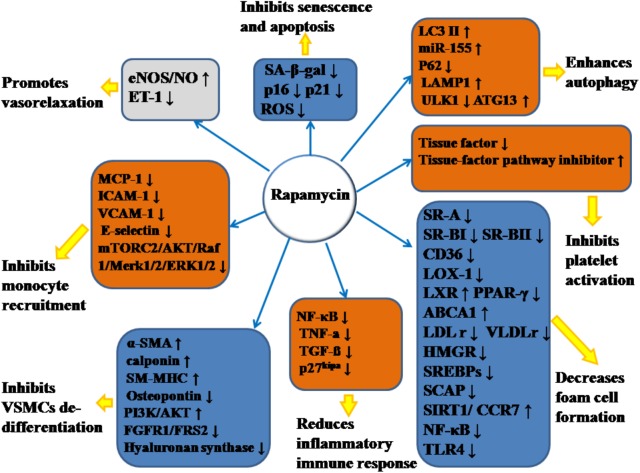
Anti-atherosclerotic effects of rapamycin and its molecular targets. Rapamycin inhibits the formation and development of atherosclerosis (AS) by promoting vasorelaxation, inhibiting monocyte recruitment, inhibiting VSMCs de-differentiation, reducing inflammatory immune response, decreasing foam cell formation, inhibiting platelet activation, enhancing autophagy, and inhibiting senescence and apoptosis. The notation ↑ indicates increase or activation, and ↓indicates decrease or suppression. LOX-1, lectin-like oxidized low-density lipoprotein-1; SR-A, scavenger receptor, class A; SR-BI, scavenger receptor, class B, type I; SR-BII, scavenger receptor, class B, type II; LAMP1, lysosome-associated membrane protein 1; LC3, light chain 3; eNOS, endothelial nitric oxide synthase; NO, nitric oxide; ET-1, endothelin-1; MCP-1, monocyte chemotactic protein-1; LXRa, liver-X-receptor alpha; PPAR-γ, peroxisome proliferator activated receptor gamma; ABCA1, ATP-binding cassette transporter A1; TLR4, toll-like receptor 4; ROS, reactive oxygen species; SM-MHC, smooth muscle myosin heavy chain; FGFR1, fibroblast growth factor receptor-1; FRS2, fibroblast growth factor receptor substrate 2; SA-β-gal, senescence-associated galactosidase; ATG13, autophagy-related protein-13; LDLr, low-density lipoprotein receptor; VLDLr, very low-density lipoprotein receptors; HMGR, 3-hydroxy-3-methylglutaryl coenzyme A reductase; SREBPs, sterol regulatory element-binding proteins; SCAP, SREBP cleavage-activating protein; TGF-β, transforming growth factor-β; ICAM-1, intercellular adhesion molecule-1; VCAM-1, vascular cell adhesion molecule-1; NF-κB, nuclear factor-kappa B; miR-155, microRNA-155; SIRT1, sirtuin 1; ULK1, Unc-51 like autophagy activating kinase 1; α-SMA, α-smooth muscle actin; CCR7, C-C chemokine receptor type 7; CD36, cluster of differentiation; TNF-α, tumor necrosis factor-α; mTORC2, mTOR complex 2.

Emerging evidence has demonstrated that RAPA has pleiotropic protective function against AS and therefore may have therapeutic value. In animal models, RAPA decreased plaque burden (Elloso et al., 2003; Castro et al., 2004; Pakala et al., 2005; Gadioli et al., 2009; Zhao et al., 2009; Liu et al., 2016; Ma et al., 2017; Zhang et al., 2017). It also enhanced the stability of plaque (Chen et al., 2009; Luo Z. et al., 2017; Ma et al., 2017), which was characterized by increased fibrous cap content, decreased necrotic core, and attenuated inflammation of plaque (summarized in Table 1). The anti-AS mechanisms of RAPA are elaborated through anti-inflammatory immune, lipid-modulatory, apoptosis and autophagy, and anti-thrombotic effects in multiple cellular models that simulate early and advanced AS formation. Anti-AS effects of RAPA and its molecular targets are summarized in Figure 2.

**Table 1:** Details of animal experiments investigating the effects of rapamycin (RAPA) in atherosclerosis (AS).

	Author and year	Animal model	Diet	Duration	Concentration	Administration	Lipid level
Decrease in AS lesion	Elloso et al., 2003	ApoE KO C57	HF	12 weeks	1, 2, 4, and 8 mg/kg	Oral	Increase in LDLc and HDLc
	Gadioli et al., 2009	ApoE KO C57	NC	12 weeks	5 mg/kg	Oral	Increase in TC
	Castro et al., 2004	ApoE KO C57	HF	6 weeks	1 and 4 mg/kg	Oral	NS
	Zhao et al., 2009	LDLR-deficient C57	HF	8 or 16 weeks	0.1, 0.3, and 1 mg/kg	Oral	NS
	Liu et al., 2016	ApoE KO C57	HF	8 weeks	Not noted	Intra-peritoneal	Not noted
	Basso et al., 2003	ApoE KO C57	HF	12 weeks	4 mg/kg	Oral	Not noted
Enhance AS stability	Chen et al., 2009	Rabbit	HC	20 weeks	0.5 mg/kg	Oral	NS
	Ma et al., 2017	ApoE KO C57	HF	8 weeks	0.5 mg/kg	Oral	Decrease in lipid levels
Decrease in AS lesion and enhance AS stability	Luo Z. et al., 2017	ApoE KO C57	HF and HC	16 weeks	50 and 100 mg/kg	Oral	Not noted
	Dou et al., 2016	ApoE KO C57	HF	12 weeks	1 and 3 mg/Kg	Subcutaneously	Increase in LDLc
Decrease eNOS expression	Cheng et al., 2008	ApoE KO C57	HC	3–4 weeks	3 μg/kg, 3 mg/kg	Oral	Not noted


*HF, high fat diet; NC, normal chow diet; HC, high cholesterol diet; LDLc, LDL-cholesterol; HDLc, HDL-cholesterol; TC, total cholesterol; NS, non-significant; AS, atherosclerosis*.

### Ameliorating Endothelial Function and Inhibiting Monocyte Recruitment

Endothelial cells, as the components that are directly in contact with circulating blood, are important in maintaining vascular homeostasis. Impaired endothelial function is an initiating event in the formation of AS (Roth et al., 2015). RAPA can improve endothelial function through regulating NO expression. Shear stress can affect the production of NO via the shear stress-responsive eNOS promoter, and low-stress regions are prone to AS (Cheng et al., 2008). In carotid arteries of mice fed normal- or high-cholesterol diet, RAPA modulates shear stress-induced NO production. RAPA can upregulate intracellular eNOS expression at low and normalshear-stress levels induced by carotid cast placement, an observation that indicates that the administration of RAPA can retard the onset of AS at AS-prone sites through increasing NO production (Cheng et al., 2008). Additionally, Trapp and Weis (2005) have reported that RAPA increased the production of NO in endothelial cells dose-dependently *in vitro*. Even in hypoxia, when NO production is greatly reduced, RAPA can increase the production of NO in endothelial cells. On the other hand, endothelin-1 is released by endothelial cells, which promotes vasoconstriction and enhances the adhesion of immune cells to endothelial cells. RAPA can decrease the production of endothelin-1 dose-dependently, thereby inhibiting the vasoconstriction of endothelial cells (Guo et al., 2014). Thus, RAPA can promote vasorelaxation through increasing NO production and inhibiting vasoconstriction of endothelial cells through targeting cytokine secretion.

In addition to endothelial impairment, monocyte recruitment is the hallmark of early AS. Recent evidence has also demonstrated that RAPA reduces adhesion of monocytes to endothelial cells. The migration of monocytes is increased after exposure of stromal cell-derived factor-1, a chemoattractant for monocyte recruitment. Pretreatment of monocytes with RAPA can inhibit this chemotaxis dose-dependently. This activity of RAPA is consistent with the results of an *in vivo* study in which macrophage numbers in plaque were decreased in RAPA-fed atherogenic mice (Pakala et al., 2005). Monocyte chemotactic protein-1 also participates in recruiting monocytes. RAPA has been found to attenuate monocyte chemotactic protein-1 expression in the aortic arch of apoE-deficient mice fed an atherogenic diet, a finding that illustrates that RAPA has anti-migratory effects on macrophages (Castro et al., 2004). Additionally, RAPA can dose-dependently decrease the ox-LDL-induced expression of ICAM-1 and E-selectin and inhibit the adhesion of monocytes to human umbilical vein-endothelial cells through suppressing the activation of MTORC2, and then inhibiting PKC phosphorylation and c-fos expression (Sun et al., 2017). Moreover, TNF-α, an inflammatory cytokine, can drive adhesion molecule expression through activating the Raf-1-Merk1/2-ERK1/2 pathway (Roberts and Der, 2007). It has been reported that RAPA can reduce increased TNF-a-induced expression of VCAM-1 by inhibiting the mTORC2-AKT-Raf-1-Merk1/2-ERK1/2 pathway in human umbilical vein-endothelial cells (Wang et al., 2014). However, RAPA had no effect on adhesion molecules expressed in human macrovascular and microvascular endothelial cells, which seems to contradict results of the above phenomenon (Lehle et al., 2008); the inconsistent results might be due to variations in physiological properties of the above three types of endothelial cells.

### Modulating the Phenotypic Switch of Vascular Smooth Muscle Cells

Following the endothelial injury, VSMCs carry out a phenotypic transition from differentiation to de-differentiation and migrate to the sub-endothelial layer, where they and proliferate quickly, forming neointima. RAPA inhibits the de-differentiation of VSMCs through inhibiting mTOR and S6K1, resulting in changes in cellular contractile morphology and increased waf-1 and p27^kip^, a reaction that results in cell-cycle withdrawal (Ma et al., 2007). In a relevant *in vitro* study (Martin et al., 2004), RAPA prompted VSMCs to assume a contractile phenotype by inducing α-smooth muscle actin (α-SMA), calponin, and SM-MHC at the mRNA and protein levels through targeting S6K1. Also, in freshly excised swine femoral arteries, the contractile protein series, previously down-regulated by normal organ culture, was reversed after treatment with RAPA (Ding et al., 2011). Additionally, during the phenotypic transition of VSMCs, various growth factors or receptors, such as PDGF, IGF, and FGFR, are involved. When VSMCs are incubated with PDGF (Pan et al., 2017), RAPA can inhibit the proliferation and migratory effects of VSMCs together with increasing α-SMA and calponin and downregulating osteopontin protein; IGF is universally distributed in the blood serum, and the IGF-I receptor can be expressed in insulin-sensitive tissue and VSMCs. After binding with IGF, the IGF-I receptor can activate the PI3K-Akt pathway, which is a critical mechanism for maintaining the contractile phenotype of VSMCs (Ohkawa et al., 2003). RAPA can enhance the contractile protein expression via the IGF-I-PI3K-AKT pathway. FGFR1, as the major form of FGFR with its downstream signaling pathway, participates in VSMCs phenotypic switching through modulating contractile marker gene expression. FRS2 acts as a downstream modulator of the FGFR-1 pathway and, after FGFR-1 binds with FGF, FGFR-1 combines with FRS2 to form FGFR-1/FRS2 complex. The complex combines with mTOR to form multi-protein composites, followed by the activation of mTOR, and inhibits the contractile marker expression. RAPA can reverse FGFR-1/FRS2/mTOR complex-mediated downregulated expression of the contractile marker gene (Chen and Friesel, 2009).

**FIGURE 3:**
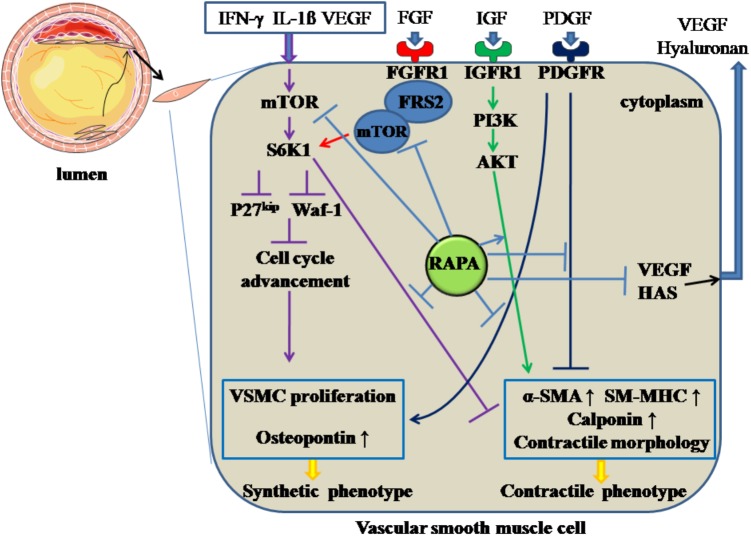
Rapamycin inhibits the de-differentiation of vascular smooth muscle cells. Rapamycin suppresses the de-differentiation of vascular smooth muscle cells by inhibiting the activation of mTOR and S6K1, activating PI3K/AKT pathways, inhibiting PDGF pathway and the formation of FGFR-1/FRS2/mTOR complex. In addition, rapamycin inhibits the synthesis of hyaluronan through decreasing HAS and the secretion of VEGF. The solid lines with arrow and notation ↑ indicate increase or activation, and lines without arrows indicate decrease or suppression. VSMC, vascular smooth muscle cell; S6K1, ribosomal protein S6 kinase 1; mTOR, mammalian target of rapamycin; IFN-γ, interferon-γ; IL-1β, interleukin-1β; VEGF, vascular endothelial growth factor; RAPA, rapamycin; HAS, hyaluronan synthase; α-SMA, α-smooth muscle actin; SM-MHC, smooth muscle-myosin heavy chain; FGFR1, fibroblast growth factor receptor 1; FRS2, fibroblast growth factor receptor substrate 2; FGF, fibroblast growth factor; IGF, insulin-like growth factor; PDGF, platelet-derived growth factor.

Hyaluronan, a major element of extracellular matrix, can modulate local inflammatory responses, monocyte-VSMC adhesion, and proliferation and migration of VSMCs (Chung et al., 2002). RAPA can decrease the amount of hyaluronan secreted by VSMCs without accelerating the degradation rate of hyaluronan by decreasing the expression of hyaluronan synthase 1, 2 and 3 (Goueffic et al., 2007). In addition, RAPA-reduced hyaluronan synthesis may abolish the recruitment and retention of circulating monocytes after angioplasty, as hyaluronan can attract monocytes into inflamed tissues and promote differentiation of the cells. On the other hand, the increased proliferation of VSMCs can promote intimal hyperplasia and restenosis. The inflammatory mediator interferon (IFN)-γ has pro-AS effects, partly through promoting intimal hypertrophy and proliferation of VSMCs (Koh et al., 2004). The responsible mechanism may be induction of S6K1 phosphorylation by IFN-γ through activation of PI3K (Wang et al., 2007). RAPA can inhibit IFN-γ-mediated neointimal formation and proliferation of VSMCs by diminishing the phosphorylation of S6K1 (Wang et al., 2007). Also, VEGF can induce the proliferation of VSMCs, which have a pro-AS effect (Wang et al., 2007). RAPA has attenuated secretion of VEGF in VSMCs, thereby inhibiting the VSMC proliferation (Dichtl et al., 2006). All the above effects facilitate RAPA-mediated anti-proliferative effects in VSMCs. The above mechanisms are summarized in Figure 3.

### Modulating Lipid Metabolism

Foam cells, the typical pathological feature in AS, are derived from endothelial cells, macrophages and VSMCs. Decreasing the uptake, enhancing the degradation of ox-LDL in these cellular types is a potent mechanism for inhibiting the development of AS. In endothelial cells, 70% of ox-LDL uptake was found regulated by LOX-1. OX-LDL can induce the expression of LOX-1 through activating the NF-κB pathway and enhancing the engulfment of OX-LDL in endothelial cells (Ling et al., 1997). These effects can be reversed by RAPA, as RAPA can reduce the endocytosis of OX-LDL in endothelial cells through inhibiting mTOR phosphorylation and then decreasing IκBα phosphorylation and cellular-nuclear accumulation of P65, thereby suppressing the expression of LOX-1 (Zhou et al., 2016). RAPA can also down-regulate the expression of SR-BI, which is also required for endothelial cell-mediated OX-LDL and HDL uptake, through inhibiting mTORC1 activity (Fruhwurth et al., 2014).

On the other hand, RAPA can also modulate the processes of cholesterol uptake and efflux in macrophage. In the THP-1 cell line, SR-A and SR-BII were down-regulated in a dose-dependent manner by RAPA at the mRNA level, regardless of the duration of stimulation. CD36 and LOX-1 are especially down-regulated by high-dose RAPA stimulation over an extended period, but not in a dose- or time-dependent manner. Moreover, low-dose RAPA can increase the transcriptional level of LXRα time-dependently. High-dose RAPA can decrease PPAR-γ expression; however, this effect is attenuated over an extended time. ABCA1 expression was enhanced by a low dose of RAPA at all time periods; however, a high dose of RAPA over a long time resulted in an undetectable level of ABCA1 (Mathis et al., 2007). Also, activation of LXRα can partly induce expression of CCR7, which is needed during transport of cholesterol out of atherosclerotic plaque (Feig et al., 2010). SIRT1 can activate LXR and inhibit the NF-κB pathway, thereby performing its anti-atherogenic role (Zeng et al., 2013). RAPA can activate the SIRT1/LXR/CCR7 pathway in the U937 cell line, thereby inhibiting foam-cell formation and promoting foam-cell egress. Furthermore, by increasing SIRT1, which can inhibit activation of NF-κB, RAPA can decrease the activity of NF-κB. Activation of NF-κB decreases ABCA1 and LXRα expression, which contributes to accumulation of lipid in macrophages (Zheng et al., 2017). Moreover, the TLR4-mediated pathway can promote the formation of macrophage-derived foam cells by inhibiting the LXR pathway and ABCA1. After treating THP-1 with OX-LDL, the expression of TLR4 was markedly enhanced, mediated by the phosphorylation of mTOR and elevated p70-S6K levels in foam cells. RAPA can block the increased TLR4 induced by OX-LDL and reverse the decreased ABCA1 induced by TLR4 when its ligands are bound, thereby enhancing cholesterol efflux (Yu et al., 2011).

**FIGURE 4:**
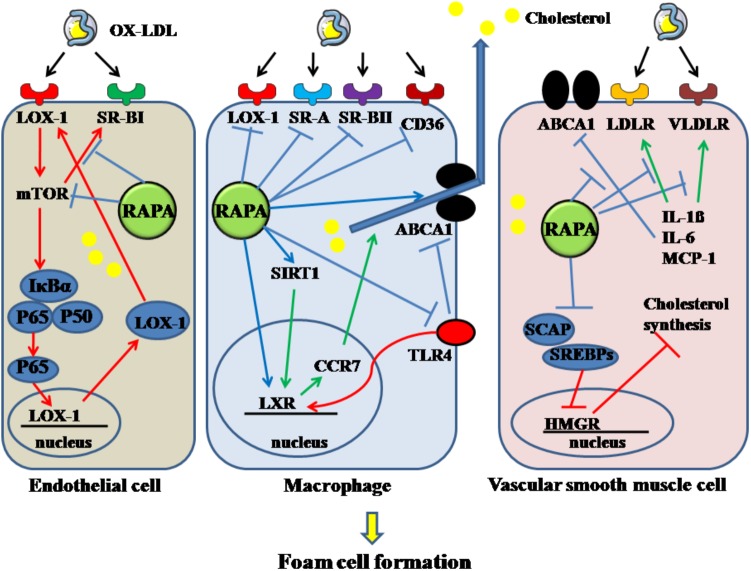
Rapamycin inhibits the formation of foam cells derived from endothelial cell, macrophage and VSMC. Rapamycin suppresses the foam cells by inhibiting the activation of mTOR, SR-BI in endothelial cells. It decreases macrophage-derived foam cells by inhibiting LOX-1, SR-A, SR-BII, CD36, TLR4 and enhancing the expression of LXR, SIRT1, and ABCA1. In addition, it decreases VSMC-derived foam cells by elevating the decreased ABCA1 induced by IL-1β, IL-6, MCP-1, and downregulating the increased LDLR and VLDLR. And rapamycin can also decrease cholesterol synthesis. The solid lines with arrow indicate increase or activation, and lines without arrows indicate decrease or suppression. OX-LDL, oxidized low-density lipoprotein; LOX-1, lectin-like oxidized low-density lipoprotein-1; SR-A, scavenger receptor, class A; SR-BI, scavenger receptor, class B, type I; SR-BII, scavenger receptor, class B, type II; CD36, cluster of differentiation 36; RAPA, rapamycin; ABCA1, ATP-binding cassette transporter A1; TLR4, toll-like receptor 4; VLDLR, very low-density lipoprotein receptor; LDLR, low-density lipoprotein receptor; mTOR, mammalian target of rapamycin; SIRT1, sirtuin 1; CCR7, C-C chemokine receptor type 7; LXR, liver-X-receptor; HMGR, 3-hydroxy-3-methylglutaryl coenzyme A reductase; SREBPs, sterol regulatory element-binding proteins; SCAP, SREBP cleavage-activating protein; IL-1β, interleukin-1β; MCP-1, monocyte chemotactic protein-1.

Rapamycin can inhibit the formation of VSMCs-derived foam cells. Their lipid content can be increased through increased LDL receptor-guided LDL uptake or reduced ABCA1-guided cholesterol efflux, induced by inflammatory cytokines. RAPA can decrease the increased expression of LDL and VLDL receptors, increased by IL-1β, resulting in a reduction of intracellular lipid contents in VSMCs. In addition, RAPA enhanced cholesterol efflux and reversed decreased cholesterol efflux in the inflammatory environment caused by IL-1β via elevating ABCA1 and ABCG1 expression (Ma et al., 2007). Moreover, RAPA can attenuate the expression of IL-6, IL-8, monocyte chemotactic protein-1, which can promote foam-cell formation (Ma et al., 2007). RAPA cannot only decrease the uptake of LDL by VSMCs, it can inhibit cellular cholesterol synthesis, which is enhanced by inflammatory stress. Cholesterol synthesis is limited by a key enzyme, HMGR. When synthesis is demanded, SREBPs are transported via SCAP from the endoplasmic reticulum to the Golgi and activated by SCAP in the Golgi, followed by translocation into the nucleus and binding with the promoter of HMGR. In this circumstance, cholesterol synthesis is activated. When cholesterol is overloaded in cells, the insulin-induced gene, a membrane protein located in the endoplasmic reticulum, connects with SCAP to inhibit the activation of SREBPs, which form a feedback loop for cholesterol homeostasis. In addition, the insulin-induced gene mediates HMGR degradation via the ubiquitin-proteasome pathway. Normal function of the feedback loop is essential for inhibiting foam-cell formation. With inflammatory stimulation, such as with IL-1β, the feedback loop is disrupted and cholesterol synthesis in VSMCs is increased. In the presence or absence of IL-1β, RAPA can inhibit cholesterol synthesis in VSMCs through sequentially downregulating the expression of SCAP, SREBP-2, and HMGR, upregulating Insigs, and decreasing nuclear translocation of SREBP-2/SCAP composite (Ma et al., 2010). Thus, foam cells derived from VSMCs can be inhibited by RAPA through targeting cholesterol uptake, efflux and synthesis. The above mechanisms are summarized in Figure 4.

### Inhibiting Inflammatory Immune Response

In advanced lesions, inflammatory immune responses, including innate and adaptive reactions, exacerbate cellular apoptosis and death, thereby decreasing plaque stability. RAPA can ameliorate cellular innate inflammatory responses. Pathogen-associated molecules derived from bacteria, viruses or fungi, can bind with TLR4, leading to triggering of the inflammation cascade. RAPA can down-regulate inflammatory cytokines, such as IL-6 and TNF-a, in THP-1 macrophages treated with LPS (Varghese et al., 2005). Additionally, in the proinflammatory pathway, NF-κB, a transcription factor, is a main mediator of cytokine secretion to promote inflammation and recruit other immune cells in AS. RAPA can decrease NF-κB activity, possibly through increasing SIRT1, which is characterized by decreased levels of NF-κB in the nucleus and its downstream target, TNF-α (Zheng et al., 2017). On the other hand, RAPA can suppress the proliferation of T-cells that may contribute to AS, and both T helper type 1 and T helper type 2 cytokine production are decreased by treatment with RAPA. Moreover, RAPA can increase mRNA expression of TGF-β, which retards the proliferation of T cells (Dumont and Su, 1996). Mechanistically, RAPA attenuates cell-cycle advancement through inhibitory regulation of the CKI p27^kipa^. In another study, RAPA has been demonstrated to increase the expansion of Treg cells, a finding that indicates that RAPA can modulate the adaptive immune response to inhibit AS (Potekhina et al., 2011).

### Inhibiting Cellular Senescence and Apoptosis

Cellular senescence and apoptosis have been commonly observed in advanced lesions and can increase plaque vulnerability. It is well established that senescent VSMCs have impaired self-repairing ability, which reduces fibrous cap content (Luo Z. et al., 2017). OX-LDL can induce the senescence of VSMCs, and SA-β-gal, p16 and p21 have been found increased in senescence VSMCs. RAPA decreased SA-β-gal, p16 and p21 expression in VSMCs, an activity that indicates that RAPA attenuates cellular senescence increased by OX-LDL (Luo Z. et al., 2017).

The apoptosis of macrophage and VSMCs induces plaque vulnerability in advanced plaque (Clarke et al., 2006). The apoptosis rate of macrophage-derived foam cells varies with cellular stage, which is determined by the duration of stimulation with OX-LDL. In THP-1 treated with OX-LDL, foam-cell assay was performed at 6 and 48 h, which represent early and late stages of foam-cell formation (Liu et al., 2016). At both times, the intracellular lipid content was significantly increased, and the transformation of LC3I to LC3II was upregulated. Foam cells at late stage are prone to apoptosis because of increased oxidative stress, but upon treatment with RAPA, the viability of late-stage cells is increased (Liu et al., 2016). Also, in the same paper, after feeding mice with a RAPA-included western diet for 16 weeks, aortic lesions had a lower apoptotic cell profile than that did controls, and the predominant cell type that colocalized with autophagy markers was macrophage (Liu et al., 2016). These results indicate that RAPA can prevent plaque advancement of AS and enhance plaque stability through repressing the apoptosis rate of foam cells at various stages. Mechanistically, RAPA decreases apoptosis through suppressing the production of ROS, which promote cellular apoptosis, thereby inhibiting creation of mitochondria-derived superoxide.

### Stimulating Autophagy

Autophagy is a cellular protective process, in which damaged intracellular elements, such as dysfunctional mitochondria, proteins, and lipid droplets, are eliminated, a process that contributes to cellular balance and self-adaptation (Yang and Klionsky, 2010). Autophagy has been classified into three categories: Microautophagy, macroautophagy, and chaperone-mediated autophagy (De Meyer et al., 2015). The main steps of macroautophagy are autophagosome formation, cargo segregation, and, finally, lysosomal fusion/degradation. Impaired macroautophagy can lead to inflammatory diseases, such as AS (Levine et al., 2011). In advanced AS, dysfunctional macroautophagy can make cells vulnerable to apoptotic stimuli and impaired clearance of apoptotic cells, which accelerates plaque progression (De Meyer et al., 2015; Grootaert et al., 2018). Therefore, interest in agents that can stimulate autophagy has increased, and RAPA has been found to have macroautophagy-stimulating effects (Adelman, 2010). In the constricted carotid artery of ApoE-/- mice, created by collar surgery, oral administration of RAPA increased the formation of autophagosomes and activated autophagy, thereby diminishing plaque development; the responsible mechanism is: RAPA increases miRNA-155 expression, thereby promoting the activation of autophagy, which retards the development of atherosclerotic plaques (Ma et al., 2017). Moreover, low-dose RAPA, as a moderate stimulator of autophagy, can reverse the decreased VSMC autophagy induced by OX-LDL through inducing increased autophagosome and elevated expressions of LC3II. In this process, activated mTORC1 can induce the phosphorylation of Unc-51-like autophagy, thus activating kinase 1 (ULK1) and repressing ULK1 and ATG13 expression; through these actions, ULK1/ATG13 complex-induced autophagy is inhibited. After RAPA treatment, MTORC1, ULK1 total content, and phosphorylated ULK1 were decreased, and ATG13 was upregulated, whereby VSMCs autophagy survival was enhanced (Luo Z. et al., 2017). Intriguingly, with increased doses of RAPA, its anti-AS effects were not increased, which implies that moderate autophagy induced by low-dose RAPA – without inducing excessive autophagy – may be optimal for treatment of AS.

On the other hand, induced by OX-LDL, autophagy in endothelial cells is increased, and autophagosomes and autolysosomes are formed, followed by an infusion with lysosome, by which OX-LDL is degraded. RAPA can enhance the elevated autophagy level induced by OX-LDL and decrease the accumulation of OX-LDL in endothelial cells within a certain period, thereby protecting cells against apoptosis and other damage induced by OX-LDL (Zhang et al., 2010). An *in vitro* study has also demonstrated that RAPA increased the co-localization of OX-LDL and autophagy-related proteins, such as LC3 and LAMP1. Also, p62 expression can be down-regulated by RAPA, an action that indicates that RAPA drives autophagic flux. Simultaneously, LAMP1 protein is up-regulated by RAPA, an action that implies fusing of lysosomes with autophagic vacuoles (Zhang et al., 2015). In summary, RAPA can activate the autophagy-lysosome pathway to accelerate the degradation of OX-LDL in endothelial cells. However, after continual stimulation with OX-LDL, RAPA does not affect the endothelial content of OX-LDL, possibly because excessive OX-LDL can damage the autophagic degradation of itself (through interfering with the fusion between lysosomes and autophagosomes).

### Inhibiting Platelet Activation

Platelet activation and the ensuing thrombosis are responsible for undesirable outcomes, such as acute myocardial infarction or stroke, in advanced AS. In advanced atherosclerotic plaque, where rupture of the plaque and thrombosis often occur, macrophages can express tissue factor, a procoagulant molecule that triggers thrombosis and is increased by inflammatory-related stimulation (Ollivier et al., 2005). RAPA has anti-coagulant activities. Treating THP-1 and human circulating monocytes under LPS stimulation with RAPA can lead to a significant decrease in transcriptional level of tissue factor, thereby inhibiting tissue factor-antigen content and cell-surface-derived or total-tissue-generated procoagulant activity. RAPA inhibited LPS-induced nuclear translocation of the two complexes, p50/p65 and c-Rel/p65, to the tissue factor promoter site, thereby inhibiting tissue-factor activity. On the other hand, enhanced secretion of the tissue-factor pathway inhibitor induced by LPS in THP-1 cells was moderately inhibited by RAPA (Ollivier et al., 2005). The above effects lead to the reduction of platelet activation by RAPA.

## Clinical Application of Rapamycin in Atherosclerosis

Sirolimus-eluting stents, the first drug-eluting stent approved by the United States Food and Drug Administration, were widely used in treating AS lesions at coronary and other peripheral arteries. The stents were used in the treatment of *de novo* AS lesions, graft stenosis, and in-stent restenosis, and they had better outcomes than did PTA or other stents. The randomized controlled clinical trials comparing SES and other stents or PTA are tabulated in Table 2.

**Table 2:** Randomized controlled clinical trials comparing SES and other stents or PTA in the past 10 years.

Follow up duration	Author and year	Lesion	Cases (n)	Control group	Primary endpoint	Results
110 days	Rastan et al., 2012	*De novo* infrapopliteal artery	161	BMS	Event-free survival rate, target vessel revascularization rate	The rates significantly improved with SES in comparison with BMS
6 months	Falkowski et al., 2009	Crural arteries	25	BMS	In-stent restenosis rate	SES decreased restenosis rate in comparison to BMS
1 year	Katsanos et al., 2016	*De novo* peripheral artery	200	PTA	Complete wound closure, quality of life	SES accelerated wound healing, improved quality of life
	Lemos et al., 2015	*De novo* coronary artery	170	Biolimus-eluting stent	Target lesion revascularization rate, stent thrombosis	Non-inferior
	Scheinert et al., 2012	*De novo* peripheral artery	200	PTA	In-segment binary restenosis rate.	Lower restenosis rate for SES
	Lee et al., 2010	Graft restenosis in coronary artery	172	Paclitaxel-eluting stents	Major adverse cardiac events, target vessel revascularization rate	Paralleled clinical outcomes
10 years	Yamaji et al., 2016	Coronary artery	1012	Paclitaxel-eluting stents	Major adverse cardiac events, target lesion revascularization rate.	SES decreased target lesion revascularization rate


*SES, sirolimus-eluting stent; PTA, percutaneous transluminal angioplasty; BMS, bare metal stent*.

### Rapamycin in *de novo* Lesions

Sirolimus-eluting stents have advantages over PTA, standard stent or other drug-eluting stents for treatment of *de novo* lesions. For coronary lesions, it has been reported in a randomized controlled study (Morice et al., 2002) that, in-stent restenosis, repeated revascularization rates and long-term lumen loss were lower in patients who underwent interposition of SES than in those who were treated with standard stents. On the other hand, in below-the-knee atherosclerotic lesions, application of SES can contribute more to lower in-stent restenosis and target-lesion revascularization than do standard stents or PTA in randomized controlled studies (Falkowski et al., 2009; Rastan et al., 2011; Katsanos et al., 2016). Another randomized, multicenter study also demonstrates that at a mean follow-up of 33 months, compared to bare metal stents, SES improved Rutherford-Becker class, long-term event-free survival and amputation rates, but they did not significantly change target-lesion revascularization after being used to treat focal below-the-knee AS (Rastan et al., 2012). Moreover, in another randomized study (Scheinert et al., 2012), a 12-month follow-up found lower in-stent restenosis and greater vessel patency but no significant change in target-lesion revascularization, limb amputation rate, or Rutherford class level with SES versus PTA for symptomatic infrapopliteal lesions. That study also found that event-free survival was significantly higher with SES than with PTA, and SES-treated patients had a better rate of wound healing, health-associated quality-of-life scores, and quality-adjusted life-years at 12 months. Also, a 5-year retrospective analysis of SES -treated chronic below-the-knee lesions (Rutherford categories 3–6) found that although Rutherford classification and vascular patency had decreased significantly at follow-up, the patency rate was 83.8% and Rutherford class level had improved in 92% of the patients with critical limb ischemia. These results have encouraged longer follow-up with SES treatment of critical limb ischemia in patients with below-the-knee lesions (Werner et al., 2012). However, for superficial femoral artery lesions, a randomized, double-blinded, multi-center study demonstrated that at 6 and 24 months’ follow-up, SES had no significant advantage over bare metal stents in relief of symptoms, rates of restenosis, ankle-brachial indices and target-lesion revascularization rate (Duda et al., 2002, 2006). These findings indicated that the effects of SES vary among vessel segments, although these stents had favorable results after intermediate or long duration.

Sirolimus-eluting stents have advantages over other drug-eluting stents, with less in-stent restenosis and target-lesion revascularization. The commonly used agents for coating stents include sirolimus and paclitaxel. A multi-center trial found that SES and paclitaxel have similar target-lesion revascularization. At 12-month follow-up, there was no significant difference in major adverse cardiac events or stent thrombosis between the two groups (Lee et al., 2010). In another randomized controlled comparison of SES and paclitaxel in treating coronary lesions, in a follow-up of 10 years, SES had a lower rate of in-stent restenosis and target-lesion revascularization than did paclitaxel (Yamaji et al., 2016). The inconsistent results may be attributed to variation in patient or lesion characteristics. Therefore, updated meta-analysis studies should be performed to determine the long-term outcome of SES treatment compared with control treatment.

### Rapamycin in Restenosis Lesions

Saphenous vein graft interposition is commonly performed with coronary artery bypass grafting. However, the incidence of graft restenosis is significant, and restenosis often necessitates repeated revascularization (Goldman et al., 2004). Endovascular procedures with stent implantation can also be accompanied by a significant rate of restenosis. SES can be used in graft or in-stent restenosis. It has been reported that bare metal stents or PTA have a high restenosis rate compared with that of SES in treating saphenous vein graft lesions (Brilakis et al., 2009). Moreover, in a cohort of 273 patients with 364 coronary lesions, including graft or in-stent restenosis, SES were used, and the mean 24-month follow-up revealed rates of binary restenosis, target-lesion revascularization and stent thrombosis of 5.5, 4.7, and 0.3%, respectively, which are lower than those reported with bare metal stents (Ino et al., 2009).

## The Main Challenge to the Application of Rapamycin

The clinical application of RAPA can be accompanied by side effects that limit the routine application of RAPA in patients with AS. The most frequent complication is hyperlipidemia, which may develop in transplant patients treated with RAPA (Kniepeiss et al., 2004). The elevated lipid profiles caused by RAPA may accelerate the formation of AS. The explanation for the hyperlipidemia may be that RAPA suppresses LDL receptor expression in the liver, thereby elevating blood cholesterol and apoproteins concentration and promoting hepatic secretion of triglycerides (Andres et al., 2006). Another study (Otsuka et al., 2015) demonstrated dose-dependent hyperlipidemia induced by RAPA, which could be managed well with postoperative statin therapy. Therefore, a lipid-lowering regimen and lower dosage of RAPA may be needed for patients being treated with RAPA. Other unwanted effects of RAPA are: (1) Inhibitory effects of endothelial progenitor cell-induced re-endothelization, which may cause late stent thrombosis and sudden death. Endothelial progenitor cells, differentiated from circulating MNCs, can repair damaged endothelial cells, thereby promoting re-endothelization (Kalka et al., 2000). RAPA inhibits the differentiation of MNCs into endothelial progenitor cells and promotes the senescence of these cells through suppressing telomerase activity. Several clinical studies have indicated that the use of SES may moderately increase the rate of late stent thrombosis (Daemen et al., 2007; Stone et al., 2007). (2) Delayed re-endothelization via inhibition of VEGF expression and the proliferation of resident endothelial cells (Imanishi et al., 2006), promotes acute stent thrombosis, which is a common early adverse event in stent angioplasty. (3) Vasoconstriction at juxta-SES-implanted coronary segments, which results in the formation of AS in non-target vessels. In evaluating the endothelial function influenced by SES or bare metal stents, vasoconstriction induced by acetylcholine provocation test, as a hallmark of endothelial dysfunction, is elevated at juxta-SES-implanted coronary segments compared with that in the juxta-bare metal stents coronary segment in patients with multiple lesions (Mischie et al., 2013). This finding implies that RAPA may trigger the AS cascade, as impaired endothelial cell function initiates the formation of AS adjacent to the stented vascular segments (Bonetti et al., 2003), with more in-stent restenosis at the edge of SES. This result rules out individual variation because the vasoconstrictive effect occurs at different vascular segments in the same patient. Overall, these adverse effects can offset the promising advantages of RAPA in treating AS lesions.

In advanced atherosclerotic plaque, overactivation of autophagy commonly occurs, which results in elevated cell death or apoptosis. Excessive autophagy stimulation or defective autophagy is a deterrent to treating AS because it can have unexpected adverse events. Additionally, several studies have demonstrated that the vulnerability of various cells to external injury is increased under the above-mentioned autophagy conditions (Jung et al., 2008; Liao et al., 2012). Therefore, the adequate dosage of RAPA used to prevent AS formation or in-stent or graft restenosis should be determined with pharmacological dynamic studies of the optimal blood RAPA concentration achieved with various delivery methods and dosages of the drug. Moreover, although *in vitro* and *in vivo* studies have suggested the optimal dosage and stimulation period of RAPA for decreasing the expression of various AS-inducing genes, this information has not been translated into RAPA administration methods in clinical application. There has been much variation in animal models used, such as treatment with angioplasty, variation in vessel grafts, different genetic deficiencies, and differences in cell lines and stimuli. Therefore, determining the optimal concentration and administration period of RAPA is imperative before considering this agent as an anti-AS agent.

Novel methods for achieving safer and more effective administration of RAPA than by systemic administration are being explored. Various types of delivery systems have been developed to reduce systemic side effects in AS, including a liposomal delivery system, a nanoparticle system, and drug-loaded microbubbles (Kilroy et al., 2015; Miao et al., 2015; Dou et al., 2016). (1) RAPA-containing liposomes have been prepared *in vitro* through an ethanol injection method. The results demonstrated high encapsulation rate of RAPA in the prepared liposomes and a sustained-release effect of the *in vitro* release of RAPA (Miao et al., 2015). (2) RAPA-loaded nanoparticles, based on various acetalated β-cyclodextrin materials, have been prepared. A sustained-release effect and enhanced anti-AS activity had also been observed in an atherogenic mouse model treated with RAPA nanotherapy (Dou et al., 2016). (3) A new platform that combines intravascular ultrasound (IVUS) and drug-loaded microbubbles has been used in a swine model. Microbubble is an ultrasound contrast agent and can be loaded with RAPA; it reduced neointima formation after balloon injury in swine artery and achieved a comparable blood concentration of RAPA to a commercial SES (Kilroy et al., 2015). Although the above systems lay a foundation for a local delivery system for RAPA targeting the cellular components of atherosclerotic plaques, they have been not been used in clinical practice and need further investigation. Intriguingly, drug-eluting embolic microspheres or beads containing RAPA have been developed in transarterial chemoembolization (Fuchs et al., 2017); the effects of the system in AS remain to be elucidated.

## Conclusion

Rapamycin is a promising naturally derived compound that possesses multifaceted anti-AS mechanisms, including anti-inflammatory, lipid-modulatory, immunoregulatory, and autophagy survival effects in early and advanced AS. However, application of RAPA can have side effects, including abnormal lipid profiles, stent thrombosis, and endothelial-cell injuries. The optimal blood concentration and duration of RAPA administration for reaching and integrating the best anti-AS effects of various cell types are not known. Despite reservations about RAPA, it remains an attractive natural agent for the prevention and treatment of AS.

## Author Contributions

YL drafted the manuscript. FY and SZ searched the literature. LQ presented the topic, evaluated the literature, and checked up the manuscript.

## Conflict of Interest Statement

The authors declare that the research was conducted in the absence of any commercial or financial relationships that could be construed as a potential conflict of interest.

## Abbreviations

α-SMA: α-smooth muscle actin
4EBP-1: 4E–binding protein 1
ABCA1: ATP-binding cassette transporters A1
ABCG1: ATP-binding cassette transporters G1
AS: atherosclerosis
ATG13: autophagy-related protein-13
CCR7: C-C chemokine receptor type 7
CD36: cluster of differentiation 36
CKI: cyclin-dependent kinase inhibitor
eNOS: endothelial nitric oxide synthase
FGF: fibroblast growth factor
FGFR: fibroblast growth factor receptor
FKBP: FK-binding-protein
FRS2: fibroblast growth factor receptor substrate 2
GβL: G-protein β-subunit protein
HDL: high-density lipoprotein
HMGR: 3-hydroxy-3-methylglutaryl coenzyme A reductase
ICAM-1: intercellular adhesion molecule-1
IFN-γ: interferon-γ
IGF: insulin-like growth factor
IL: interleukin
LAMP1: lysosome-associated membrane protein 1
LC3: light chain 3
LDL: low-density lipoprotein
LOX-1: lectin-like oxidized low-density lipoprotein-1
LPS: lipopolysaccharide
LXRα: liver-X-receptor alpha
MNCs: mononuclear cells
mTOR: mammalian target of rapamycin
mTORC1: mTOR complex 1
mTORC2: mTOR complex 2
NF-κB: nuclear factor-k-gene binding
NO: nitric oxide
OX-LDL: oxidized low-density lipoprotein
PDGF: platelet-derived growth factor
PPAR-γ: peroxisome proliferator activated receptor gamma
PTA: percutaneous transluminal angioplasty
RAPA: rapamycin
ROS: reactive oxygen species
S6K1: S6 kinase 1
SA-β-gal: senescence-associated galactosidase
SCAP: SREBP cleavage-activating protein
SES: sirolimus-eluting stents
SIRT1: sirtuin 1
SM-MHC: smooth muscle-myosin heavy chain
SR-A: scavenger receptor-A
SR-BI: scavenger receptor-BI
SR-BII: scavenger receptor- BII
SREBPs: sterol regulatory element-binding proteins
SRs: scavenger receptors
TGF-β: transforming growth factor-β
TLR4: toll-like receptor 4
TNF-α: tumor necrosis factor-α
Treg: regulatory T
ULK1: Unc-51 like autophagy activating kinase 1
VCAM-1: vascular cell adhesion molecule-1
VEGF: vascular endothelial growth factor
VLDL: very low-density lipoprotein
VSMCs: vascular smooth muscle cells
